# Some like it dry: Water restriction overrides heterogametic sex determination in two reptiles

**DOI:** 10.1002/ece3.5229

**Published:** 2019-05-21

**Authors:** Andréaz Dupoué, Olivier Lourdais, Sandrine Meylan, François Brischoux, Frédéric Angelier, David Rozen‐Rechels, Yoan Marcangeli, Béatriz Decencière, Simon Agostini, Jean‐François Le Galliard

**Affiliations:** ^1^ Station d'Ecologie Théorique et Expérimentale de Moulis CNRS UMR 5321 Saint Girons France; ^2^ Centre d'Etudes Biologiques de Chize ULR CNRS UMR 7372 Beauvoir sur Niort France; ^3^ iEES Paris, Sorbonne Université, CNRS, UMR 7618 Paris France; ^4^ ESPE de Paris Sorbonne Université Paris France; ^5^ Département de biologie, Centre de recherche en écologie expérimentale et prédictive (CEREEP‐Ecotron IleDeFrance) Ecole normale supérieure, PSL Research University, CNRS Saint‐Pierre‐lès‐Nemours France

**Keywords:** dehydration, early growth, gestation, heterogamety, sex determination, sex ratio, sex reversal, survival

## Abstract

**Abstract:**

The evolution of sex determination is complex and yet crucial in our understanding of population stability. In ectotherms, sex determination involves a variety of mechanisms including genetic determination (GSD), environment determination (ESD), but also interactions between the two via sex reversal. In this study, we investigated whether water deprivation during pregnancy could override GSD in two heterogametic squamate reptiles. We demonstrated that water restriction in early gestation induced a male‐biased secondary sex ratio in both species, which could be explained by water sex reversal as the more likely mechanism. We further monitored some long‐term fitness estimates of offspring, which suggested that water sex determination (WSD) represented a compensatory strategy producing the rarest sex according to Fisher's assumptions of frequency‐dependent selection models. This study provides new insights into sex determination modes and calls for a general investigation of mechanisms behind WSD and to examine the evolutionary implications.

**OPEN RESEARCH BADGES:**

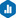

This article has earned an Open Data Badge for making publicly available the digitally‐shareable data necessary to reproduce the reported results. The data is available at https://doi.org/10.5061/dryad.mv06pv1.

## 1 INTRODUCTION

Sex determination, the process by which an individual expresses the phenotype of female, male, or both during development and adulthood, provides the cornerstone for sexual reproduction, and thus plays a key role in the maintenance of genetic diversity, dynamics of small populations and sexual selection (Le Galliard, Fitze, Ferrière, & Clobert, 2005; Kokko, Klug, & Jennions, 2012; Uller, Pen, Wapstra, Beukeboom, & Komdeur, 2007). Sex determination mechanisms vary amazingly among living species of eukaryotes and are evolutionary labile. In animals, sex can be determined at fertilization (GSD for genetic sex determination) either through heterogamety (XY males or ZW females) or polygenic sex determination (Bachtrog et al., 2014; Kraak & Pen, 2002). In many species of reptiles, amphibians and fishes, sex can also be determined by external conditions (ESD for environmental sex determination) (Bachtrog et al., 2014).

By far, ESD has been disproportionally focusing on the effects of temperature (TSD for temperature sex determination) given the broad occurrence of this strategy. In particular, reptiles are key models to study the TSD where the secondary sex ratio (i.e., ratio between males and females at birth) varies along the thermal gradient of the nest (i.e., oviparous species) or is actively controlled *via* maternal thermoregulation (i.e., viviparous species) (Robert & Thompson, 2001). In reptiles exhibiting TSD, temperature drives the sex determination during the early development of embryos through the thermo‐sensitive action of various physiological pathways such as epigenetic control of gene expression (e.g., aromatase) and hormonal secretion (Matsumoto, Buemio, Chu, Vafaee, & Crews, 2013; Pieau & Dorizzi, 2004). Although exact mechanisms of TSD remain fuzzy in many species, the evolution of TSD should be favored when the fitness of females (resp. males) is higher under thermal conditions that promote female‐biased (resp. male‐biased) sex ratios (Charnov & Bull, 1977; Pen et al., 2010; Warner & Shine, 2008).

Although most animal species have been historically classified with either GSD or ESD, both strategies are now treated as extremes of a continuum of relative influence between genotypic and environmental factors (Capel, 2017; Mank & Uller, 2014; Pen et al., 2010). For example, cases of environmental sex reversal offer striking examples of labile interactions between GSD and ESD, whereby the genetic sex is environmentally reversed during ontogeny (Stelkens & Wedekind, 2010). In reptiles for instance, high temperature during development overrides GSD in Australian bearded dragon (*Pogona vitticeps*) by feminizing ZZ males (Quinn et al., 2007), and these sex‐reversed ZZ females then performed better reproduction than heterogametic ones (Holleley et al., 2015). These findings thus place temperature sex reversal as a transient strategy which may rapidly evolve to TSD and lead to a progressive degeneration of the W or Y chromosomes in the population (Holleley et al., 2015). Many additional examples among animals showed that sex reversal might be a common strategy influenced by abiotic (temperature, pH, photoperiod, endocrine effects) or biotic (social rank, population density, parasitic *Wolbachia* sp) factors whose prevalence might accelerate with anthropogenic activities (Stelkens & Wedekind, 2010). Notably, in regards with global warming, abnormal temperature exposures can destabilize operational sex ratio (i.e., ratio between breeding females and males), potentially leading to a population extinction (Pezaro, Doody, & Thompson, 2017). Such statement illustrates the critical importance to clarify the environmental cues leading to ESD or sex reversal in understanding and predicting organisms’ responses to future climates.

Given the dominant effect of thermal conditions on the ecology and the evolution of reptiles, the role of other environmental factors in sex determination has been neglected. Together with environmental temperatures, water availability is another critical ecological factor that dramatically influences homeostasis and fitness in reptiles (Bradshaw, 1997). Water sex determination (WSD) occurs in plants (Freeman & Vitale, 1985), and in one turtle species known for exhibiting TSD, nest moisture seems to interact with thermal conditions in shaping secondary sex ratio, thereby suggesting a potential contribution of water in sex determination (Sifuentes‐Romero, Tezak, Milton, & Wyneken, 2018). Furthermore, reptiles are known to express considerable developmental plasticity in response to water conditions during embryogenesis (Packard, 1991). Contrary to temperature, water is a depreciable resource meaning that during gravidity or gestation, females have to divide water between embryos hence suggesting an additional level of variation with in utero intraclutch competition (Bonnet, Naulleau, & Shine, 2017). Maternal control of water allocation into offspring through hydroregulation mechanisms during gestation influences offspring fitness and can differentially influence sons and daughters (Dupoué et al., 2018; Le Galliard, Massot, Landys, Meylan, & Clobert, 2006). Yet, it remains unknown if changes in water availability during embryonic development can cause sex reversal.

In this study, we tested the impact of water availability during gestation on sex ratio variation in two heterogametic and viviparous (live‐bearing) reptiles, a snake (*Vipera aspis*) and a lizard (*Zootoca vivipara*). In these species, sex at birth is reported to be chromosomally determined through the ZZ‐ZW system (Aprea, Gentilli, Zuffi, & Odierna, 2006; Chevalier, Dufaure, & Lecher, 1979). We first experimentally tested whether a two‐week period of water restriction in mid‐pregnancy can cause secondary sex ratio variation beyond the 1:1 expectation in GSD species. Since water restriction protocol has previously been shown to significantly dehydrate females without modifying reproductive investment (Dupoué et al., 2015, 2018), unbalanced sex ratios could imply WSD. We further investigated whether distorted secondary sex ratios could be explained by alternative hypotheses to WSD per se including (a) differential sex‐biased mortality, (b) methodological biases, or (c) statistical artifacts. We lastly examined the potential for WSD as a selective strategy by testing the assumptions of the Charnov & Bull models for evolution of sex determination (Charnov & Bull, 1977; Warner & Shine, 2008). If water restriction increases the proportion of one sex at birth, this theoretical model predicts that fitness of this sex should be favored when produced by water restricted mothers. Given that both species are capital breeders with strong influence of body size on reproductive investment, we used offspring early body growth and annual survival rates, as reliable estimates of lifetime reproductive success (Bonnet, Lourdais, Shine, & Naulleau, 2002; Le Galliard, Clobert, & Ferrière, 2004).

## 2 MATERIALS AND METHODS

### 2.1 Capture, husbandry, and experimental design

#### 2.1.1 Species 1: the Asp viper

The Aspic viper (*V. aspis*) is a medium‐size snake (~60 cm adult body size) that is typically found in hedgerow landscapes in Western Europe. In May and June 2012, we caught 29 pregnant females from neighboring sites in western France (Vendée and Loire‐Atlantique Districts) and we assessed gestation using ultrasonography (Dupoué et al., 2015). Females were housed 3–4 per cage in 8 cages (100 × 30 × 35 cm) following the husbandry as detailed previously (Dupoué et al., 2015). Females were provided a thermal gradient (20–40°C) 5 hr per day with a 75W light bulb at one extremity of the cage and were provided with water ad libitum with four water bowls and two sprays per day. Each female was randomly assigned to either a control or a water deprivation treatment. In the control group, females were maintained in the same conditions while we removed the full access to water in the water‐deprived group for three weeks (Dupoué et al., 2015). We measured the changes in plasma osmolality during the treatment as an index of hydration state, showing that water‐deprived females faced a severe dehydration, directly related to their reproductive effort (Dupoué et al., 2015). The day of parturition, we measured all neonates and stillborn offspring and sexed each individual by everting the hemipenis. Live neonates (*n* = 125) were then individualized in plastic boxes (30 × 16 × 10 cm), kept in conditions as described previously (Dupoué et al., 2016), and measured again two weeks after birth to calculate early growth rate. They were then released together with their respective mothers at the exact capture location.

#### 2.1.2 Species 2: the Common lizard

The European Common Lizard (*Zootoca vivipara*) is a small (~70 mm adult body size), widespread lacertid from humid peat bog and heathland habitats across northern Eurasia. In 2015, 2016, and 2017, we caught 420 adult pregnant females from 24 outdoor enclosures (10 × 10 m) at the Centre de Recherche en Ecologie Expérimentale et Prédictive (Saint‐Pierre‐lès‐Nemours, France, 48°17′11.42N, 2°40′46.00E). The day of capture, each lizard was identified with a unique toe clip code and maintained in conditions as previously described (Dupoué et al., 2018). Lizards were individually housed in terraria (25 × 15 × 16 cm) containing a shelter, peat soil as substrate and opportunities for optimal thermoregulation with a thermal gradient (23–38°C) 9 hours per day created by suspending a 25 W light bulb over one end of the terraria. All lizards had access to a standardized mass (400 ± 20 mg) of crickets (*Acheta domesticus*) every three days. Lizards had ad libitum access to a water bowl, and terraria were sprayed with water three times per day. At the time when pregnant females were at mid‐gestation (late May‐early June), they were randomly assigned to two experimental treatments following a previously established procedure (Lorenzon, Clobert, Oppliger, & John‐Alder, 1999). In the water restricted treatment, we removed the water bowl and reduced the misting frequency to once in the morning. In the control treatment, females had permanent access to the water bowl and were misted three times per day. Each year, water restriction lasted for 14 days. After the period of water restriction, all individuals returned to the control water conditions, having permanent access to water in a water bowl and being misted three times per day. The day of parturition, we measured all neonates and we sexed each individual by counting the ventral scales as previously established (Lecomte, Clobert, & Massot, 1992). This method has proven successful in sex determination. At recapture the next year, when the phenotypic sex is determined with clear secondary sexual characters (e.g., hemipenises in males), we found an error rate of 8% in sex determination of juveniles at birth.

Live juveniles (*n* = 2,766) were released in outdoor enclosures the day of birth (mid‐June to mid‐July) and recaptured in early September to assess the early growth rate. On the day of capture, lizards were identified with their unique toe‐clipped code, measured, weighed, and released at the end of the day. Early growth rate was calculated as the change in SVL divided by the days between measurements, since growth rate in this species is linear during the first month life (Fitze & Le Galliard, 2008).

### 2.2 Statistical analyses

All analyses were performed using R software (version 3.2.0, R Core Team 2016, https:// www.r-project.org/). Secondary sex ratio was calculated for each litter as the ratio between the number of males and number of females and analyzed with logistic regressions including a logit link and binomial error term [package *lme4*] and *z* tests were performed [package* lmerTest*]. We tested the relationships between secondary sex ratio and water deprivation treatment, female body size and reproductive timing. Reproductive timing was retro‐calculated as the day differential between parturition date and the last day of exposure to water restriction, to estimate the embryonic developmental stage when females were exposed to water restriction. For analyses in the Common lizard, we used mixed‐effect models to include the random effects of female identity given that some females had repeated contributions between years. Moreover, models included the fixed effects of year alone or interactively with female hydric treatment.

We analyzed the juvenile early body growth rate with a linear mixed model by accounting for random effects of mother identity given the non‐independence between siblings. In lizard analyses, the random effect of mother identity was nested into the outdoor enclosure in which juveniles were released. Fixed effects included the mother hydric treatment, juvenile sex, the year and first‐ or second‐order interactive terms.

In all cases, a minimum adequate model was obtained by a backward procedure where we removed nonsignificant terms one by one starting with second‐order interactions. In the Common lizard analyses, we used mixed effects models, which can generate statistical artifact due to variation in litter size among females. We therefore tested the robustness of each term selected in the final model using a parametric bootstrap analysis [package *pbkrtest*]. In this analysis, we compared the likelihood ratio test (LRT) of the final model (i.e., large model) to model without the selected term (i.e., simple model). For each term, we generated 1,000 samples of the simple model LRT that were either larger (*p* < 0.05) or equal (*p* > 0.05) than the LRT of the large model.

Results are presented as the mean ± *SE*. In figures, secondary sex ratio was represented as proportion of males in the litter.

## 3 RESULTS

### 3.1 Water restriction causes unbalanced sex ratios

**Figure 1:**
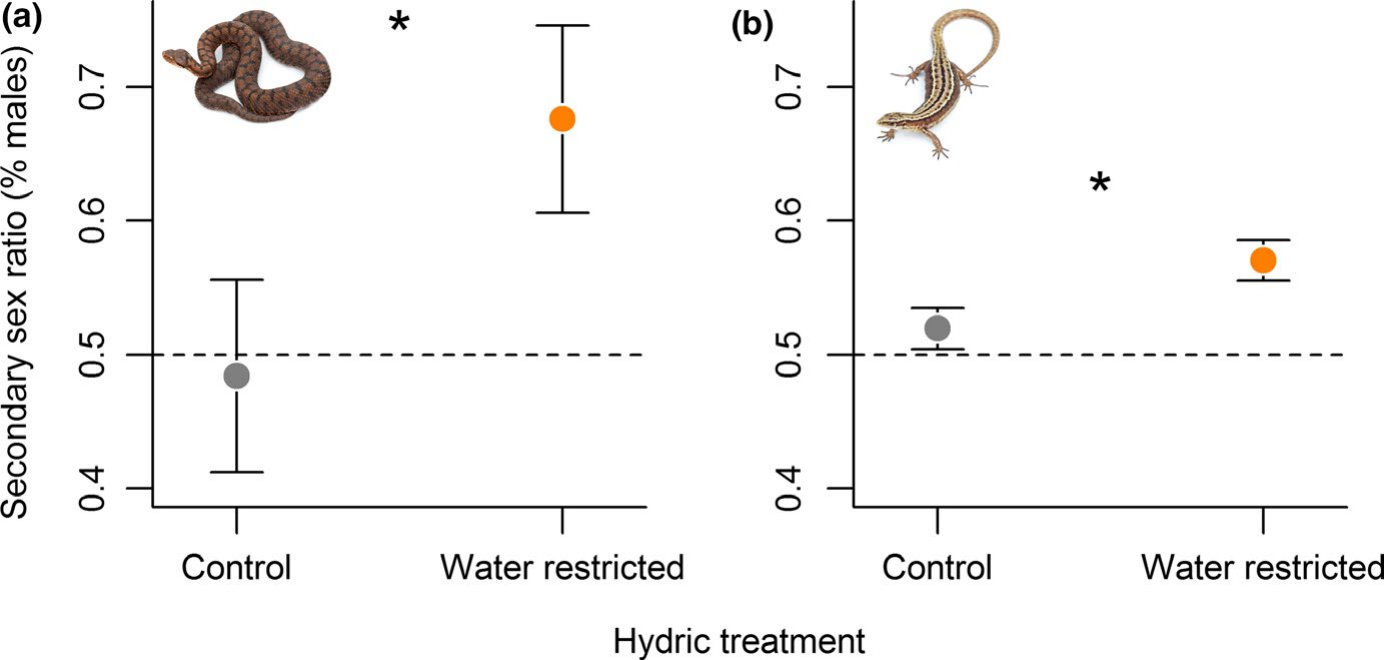
Male‐biased secondary sex ratio following water restriction in two viviparous reptile species. The average proportion of males at birth is higher in litters from water restricted pregnant females in both (a) *V. aspis* and (b) *Z. vivipara* compared to their respective controls. Data are represented as mean ± *SEM* and significant differences between female hydric treatments are symbolised: * *p* < 0.05

**Figure 2:**
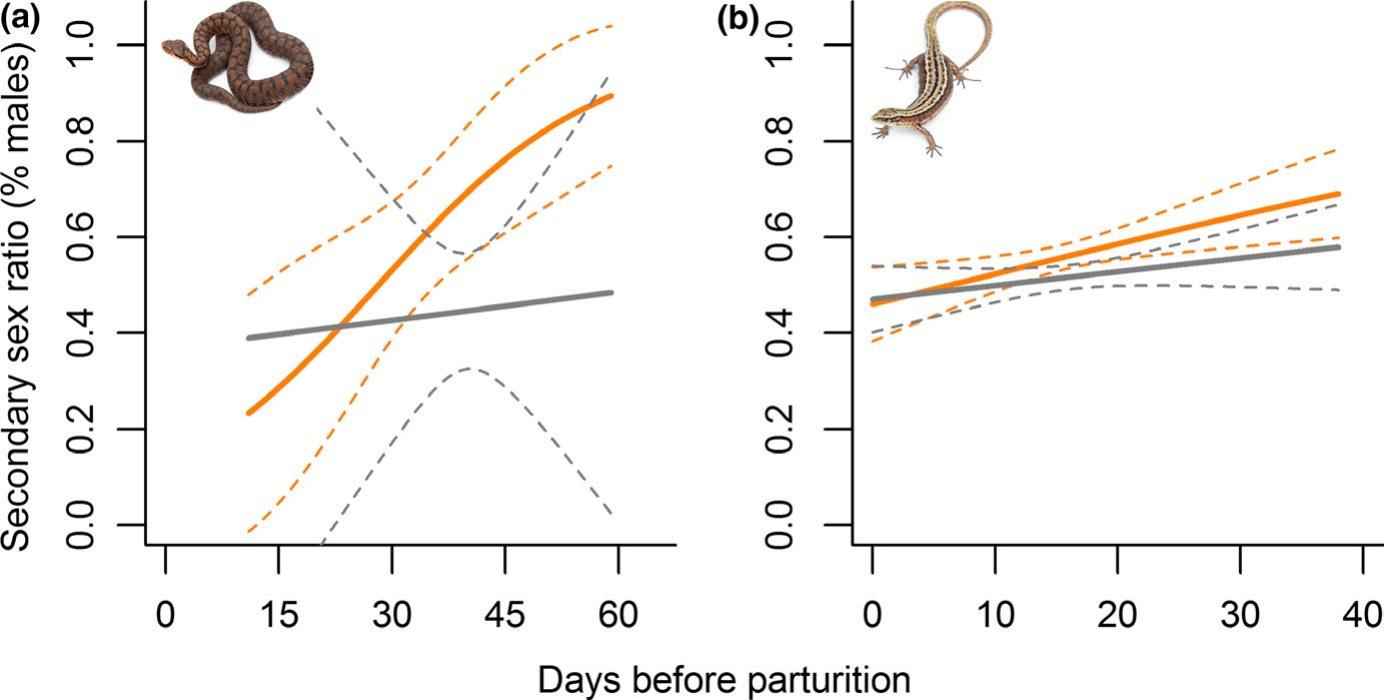
Combined effects of water treatment and reproductive timing (embryonic development) on the secondary sex ratio. We calculated the reproductive timing as the differences (days) between parturition date and the exposure to water restriction in order to estimate the embryonic developmental stage when pregnant females were exposed to water restriction (0 = parturition date). In both a) *V. aspis* and b) *Z. vivipara*, the male proportion increases when exposure to water restriction occurred earlier in gestation. The predictions of the final model were fitted on the data (solid line) together with the 95% confidence interval (dashed lines) of both control (gray lines) and water restricted females (orange lines)

In both species, water restriction during mid‐pregnancy led to a male‐biased secondary sex ratio (*vipers*: *n* = 13 females, *β* = 0.68 ± 0.28, z = 2.40, *p* = 0.016; *lizards*: *n* = 218 females, *β* = 0.26 ± 0.06, z = 4.3, *p* < 0.001), while ad libitum water availability was associated with a balanced secondary sex ratio (*vipers*: *n* = 14 females, *β* = −0.24 ± 0.25, *z* = −0.95, *p* = 0.343; *lizards*: *n* = 200 females, *β* = 0.07 ± 0.06, *z* = 1.26, *p* = 0.208). Final model retained the additive influence of the hydric treatment (Figure 1a,b, Table 1) and reproductive timing (Figure 2a,b, Table 1). That is, male‐biased sex ratio was enhanced when pregnant females were exposed to water restriction in early pregnancy, while the effects were relatively moderated in late pregnant females (Figure 2a,b). In both species, females were in the same reproductive timing during the exposition to hydric treatments (*vipers*: *t*_1,25_ = −0.8, *p* = 0.429; *lizards*: *t*_1,417_ = −0.9, *p* = 0.366). In lizards specifically, these results did not show any inter‐annual variation (between years comparisons: all *p* > 0.384) and the effects of water restriction or reproductive timing as presented above did not change between years (interactive terms between treatment and year or reproductive timing and year, all *p* > 0.289). These results demonstrate that WSD was consistent across years in this species and not an artifact of a small sample size and unique study year.

**Table 1:** AICc‐based model selection comparing the influence of body size (SVL), water restriction period (Treatment), and embryonic development (ED) on the secondary sex ratio (proportion of males and females at birth) in the aspic viper (*V. aspis*) and the common lizards (*Z. vivipara*)

*V. aspis*						*Z. vivipara*					
Model	*k*	AICc	ΔAICc	*w*i	LogLik	Model	*k*	AICc	ΔAICc	*w*i	LogLik
ED + Treatment	3	71.49	0.00	0.31	−32.22	ED + Treatment	4	1441.62	0.00	0.27	−716.76
SVL + Treatment+ED	4	72.61	1.11	0.18	−31.39	SVL + Treatment + ED	5	1441.90	0.28	0.23	−715.88
ED x Treatment	4	73.08	1.59	0.14	−31.63	ED x Treatment	5	1442.44	0.82	0.18	−716.15
SVL + ED	3	74.33	2.84	0.07	−33.64	SVL + Treatment x ED	6	1442.87	1.25	0.14	−715.33
Treatment	2	74.60	3.11	0.06	−35.08	ED	3	1445.05	3.43	0.05	−719.50
ED	2	74.97	3.48	0.05	−35.23	SVL + ED	4	1445.21	3.59	0.04	−718.55
SVL + Treatment x ED	5	75.00	3.51	0.05	−31.07	SVL + Treatment	4	1445.29	3.67	0.04	−718.59
SVL	2	75.16	3.67	0.05	−35.36	Treatment	3	1446.40	4.78	0.02	−720.17
SVL + Treatment	3	75.48	3.99	0.04	−34.28	SVL	3	1448.21	6.59	0.01	−721.07
*Null*	1	75.59	4.10	0.04	−36.72	*Null*	2	1449.43	7.81	0.01	−722.70

Note

Models were built with each factor and covariate alone and in interaction and compared to a model including the intercept only (*Null*). In lizard analyses, given the repeated design over 3 years, female identity was set as random factor. See text for details.

### 3.2 Alternative hypotheses to WSD

The first alternative hypothesis to WSD is that male‐biased sex ratio was caused by differential in utero mortality after conception and before parturition. In vipers, only 5% of neonates were stillborn (5 from control females, 3 from water restricted females) but formed enough to be sexed and therefore included in our analysis of sex ratio at birth. Moreover, thanks to an ultrasounding monitoring from ovulation to parturition, we assessed neither a loss of embryo, nor an effect of the hydric treatment on maternal water allocation nor on reproductive success at birth (Dupoué et al., 2015). Therefore, this hypothesis is not valid in vipers and sex ratio at birth provides an unbiased estimate of the secondary sex ratio. In lizards, we determined the sex of the majority of offspring including aborted, stillborn, and viable offspring (96.5% of individuals). In nonviable sex‐determined offspring (*n* = 147 late aborted fetus and stillborn), we found a similar tendency (proportion of dead males: 53.0 ± 8.1% from control mothers vs. 61.1 ± 6.3% from water restricted mothers), although nonsignificant difference between hydric treatments (*z* = 1.4, *p* = 0.163). This still shows that higher mortality of females was not the cause of male‐biased secondary sex ratios in water‐deprived litters. We further confirmed this result by performing the same statistical tests of sex ratio variation following a hypothetical scenario with a sex differential mortality. That is, in the remaining 3.5% of offspring for which sex was not determined due to uncountable scales (*n* = 110 early aborted embryos), we hypothesized female‐biased mortality of embryos by arbitrarily attributing a 1:1 male–female mortality in control mothers vs. 0:1 male–female mortality in water restricted mothers. Even under this extreme and unrealistic scenario, our results remained similar as described before hence confirming that water deprivation influenced the secondary sex ratio (Table 2).

**Table 2:** AICc‐based theoretical model selection as described in Table 1 to test the hypothesis that the male‐biased sex ratio in water restricted mothers occurred because of a female‐biased mortality

Model	*k*	AICc	ΔAICc	*w*i	LogLik
SVL + Treatment + ED	5	1474.38	0.00	0.22	−732.12
ED + Treatment	4	1474.64	0.26	0.19	−733.27
SVL + ED	4	1475.13	0.75	0.15	−733.52
ED	3	1475.48	1.09	0.12	−734.71
SVL + Treatment x ED	6	1475.76	1.38	0.11	−731.78
ED × Treatment	5	1475.90	1.51	0.10	−732.88
SVL + Treatment	4	1477.42	3.04	0.05	−734.66
SVL	3	1477.99	3.60	0.04	−735.97
Treatment	3	1479.28	4.89	0.02	−736.61
*Null*	2	1479.92	5.53	0.01	−737.94

Note

Table shows the results from model comparisons (as in Table 1) when all aborted eggs with uncountable scales have been considered only as females in water restricted mothers and randomly as males or females in the control mothers. Even under this pessimistic scenario, we found similar results than previously, hence invalidating the hypothesis of a differential mortality between sexes.

In lizards, a second alternative hypothesis to WSD would involve that hydric treatment influences the relationship between scale number and phenotypic sex, thus caused a bias in our sexing technique. To test this hypothesis, we examined data from offspring unambiguously sexed from their reproductive organs once yearling. Our sexing technique caused some errors (8% error rate). However, error rates were similar between hydric treatments (*n* = 919, *χ* ^2^_1_ = 2.86, *p* = 0.091) and the discriminant function between number of ventral scales and sex did not differ between hydric treatments (all *p* > 0.226). This demonstrates that the biased sex ratio at birth was most likely not caused by a methodological bias.

In lizards, the last alternative hypothesis implies a statistical artifact caused by the use of mixed logistic models and unequal litter size among females (Bolker et al., 2009). We therefore used a parametric bootstrap method for comparing the observed influence of hydric treatment and reproductive timing to predictions of 1,000 iterations of models under the null hypothesis of a random, balanced sex ratio. This statistically robust method retains both unambiguously the effects of hydric treatment (parametric bootstrap test: *p* = 0.02) and reproductive timing (*p* = 0.005).

### 3.3 WSD helps to maintain a balanced operative sex ratio

**Figure 3:**
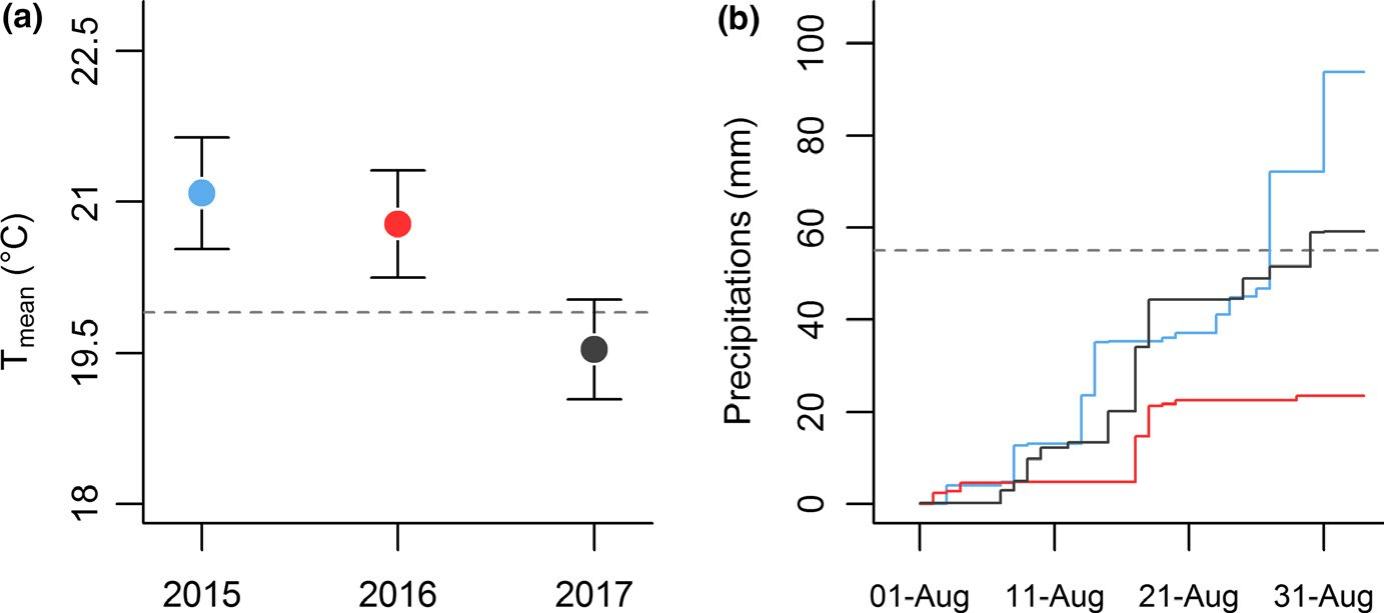
Climatic conditions registered during August in each year from the French meteorological agency. Dashed lines represent the mean of “normal” (Meteo France, http://publitheque.meteo.fr, Station 77,333,003, alt: 73m, lat: 48°16’12"N, lon: 02°42’54"E) of (a) temperature and (b) precipitation conditions during August calculated between 1981 and 2010

Our best measure of offspring fitness in both species (early body growth rate and survival) showed species‐ and sex‐specific responses. In vipers, early growth was similar between sexes (*t*_1,119 _= −0.9, *p* = 0.379, Table 3) and increased in juveniles from water‐deprived females (*t*_1,119 = _2.8, *p* = 0.009, Table 3) irrespective of juvenile sex (interaction term, *t*_1,118_ = −1.0, *p* = 0.321, Table 3). In lizards, early growth rate showed interactive effects of juvenile sex, mother treatment, and year (*t*_1,1245_ = −2.2, *p* = 0.027). Specifically in 2015, an abnormally warm and humid summer (Figure 3), maternal water restriction led to reduced juvenile female early growth rate only (Table 3). On the contrary, in 2016 and 2017 that were either respectively associated with warm and dry or normal summer conditions (Figure 3), maternal water restriction led to reduced growth rate of juvenile males only (Table 3). Eventually, this variation in early growth rate resulted in strong annual variation of juvenile survival (between years comparisons, all *p* < 0.001), and lower survival of juvenile from water restricted mothers compared to controls (*n* = 2,745, *z* = −2.7, *p* = 0.006). However, juvenile survival rate was not influenced by additive nor interactive effect of juvenile sex (all *p* > 0.105, Table 4).

**Table 3:** Annual variation of the early growth rate among juvenile females and males of Aspic vipers (*n* = 124) and Common lizards (*n* = 1,260), born from mothers exposed either to water restriction in mid‐gestation (restricted) or fully hydrated (control)

Studied species	Year	August conditions		Juvenile female					Juvenile male				
		Temperature	Precipitation	Control mothers		Restricted mothers	Statistical difference		Control mothers		Restricted mothers	Statistical difference	
*V. aspis*	2012	‐	‐	587.2 ± 42.9	<	933.0 ± 54.6	*t*_1,118 = _3.0 ; *p* = 0.005	**	621.2 ± 58.2	<	822.5 ± 46.5	*t*_1,118 = _2.2 ; *p* = 0.034	*
*Z. vivipara*	2015	Warm	High	321.4 ± 6.2	>	308.4 ± 7.2	*t*_1,1245 _= −3.0 ; *p* = 0.003	**	312.7 ± 4.6	=	311.3 ± 4.9	*t*_1,1245 _= −1.1 ; *p* = 0.267	
	2016	Warm	Low	344.2 ± 4.6	=	334.9 ± 6.1	*t*_1,1245 _= −0.3 ; *p* = 0.785		342.7 ± 5.1	>	327.2 ± 4.6	*t*_1,1245 _= −2.6 ; *p* = 0.011	*
	2017	Normal	Normal	330.5 ± 4.2	=	327.7 ± 4.2	*t*_1,1245 _= −0.8 ; *p* = 0.421		326.6 ± 3.5	>	312.6 ± 4.2	*t*_1,1245 _= −3.1 ; *p* = 0.002	**

Note

Table reports the mean (±*SEM*) of juvenile early body growth rate (in µm/day) and the differences within sex and across years between individuals born from control vs. those born from water restricted mothers, which are symbolised: *p < 0.05, **p < 0.01. Table also reports the summer climatic conditions (mean temperature and total precipitation) during August conditions (Figure 3) since juvenile lizards grew in outdoor enclosures.

**Table 4:** Annual variation of the survival rate among juvenile female and male of common lizard (*n* = 2,745 observations), born from mothers exposed either to water restriction in mid‐gestation (restricted) or fully hydrated (control)

Year	Juvenile females		Juvenile males	
	Control mothers	Restricted mothers	Control mothers	Restricted mothers
2015	20.1 ± 2.2	16.0 ± 2.3	17.0 ± 2.0	12.4 ± 1.8
2016	56.6 ± 4.2	53.6 ± 5.1	52.9 ± 4.0	51.9 ± 4.4
2017	46.6 ± 3.3	36.4 ± 3.4	45.0 ± 3.1	41.9 ± 3.2

Note

Table reports the mean (±*SEM*) of juvenile survival rate (%) within sex and across years between individuals born from control vs. those born from water restricted mothers. Survival was highly variable among years (all *p* < 0.001) and lower in juveniles born from water restricted mothers compared to those born from control mothers. See text for details.

## 4 DISCUSSION

ESD is one of the most striking examples of developmental plasticity and provides a unique framework to investigate how complex gene–environment interaction shape phenotypes and ecological responses to environmental variation (Capel, 2017; Mank & Uller, 2014; Pen et al., 2010). Here, we documented for the first time that water restriction can override heterogametic sex determination in two viviparous species known to display GSD (Aprea et al., 2006; Chevalier et al., 1979). Specifically, we found a higher proportion of males at birth in two viviparous reptiles from distinct phylogenetic families whenever pregnant females faced limitations in water availability during early gestation. We excluded the three alternative hypotheses to WSD including differential sex‐biased mortality after conception, methodological biases in sexing, or statistical artifact due to data structure and model construction. Our results, which were noticeably repeatable during three independent years in lizards, thus demonstrate the occurrence of WSD consistently with earlier studies in plants or anecdotal evidence in turtles (Freeman & Vitale, 1985; Sifuentes‐Romero et al., 2018). At the same time, our data indicated that the effect size of water deprivation was generally low on average, especially in lizards where we found an average 6% male‐bias induced by water deprivation. This suggests that water restriction may have caused sex reversal by masculinizing some but not all ZW females and/or that water deprivation had large effects only during a critical embryonic stage (see below).

The two study species invest energy into egg production prior to gestation during vitellogenesis because they are both characterized by lecitotrophic placentation. Maternal effort during gestation is thus mainly oriented to hormone or salt exchange with embryos, regulation of water supply, and thermoregulation (Dupoué et al., 2018; Le Galliard, Bris, & Clobert, 2003; Lourdais, Lorioux, Dupoué, Wright, & DeNardo, 2015). One may question the direct link between maternal water restriction and sex determination in these species since water restricted reptiles often decrease their thermal preferences or their basking activity to lower dehydration rates (Ladyman & Bradshaw, 2003; Lorenzon et al., 1999). However, earlier and independent experiments conducted in both *V. aspis* and *Z. vivipara* demonstrated that exposure of pregnant females to different thermal regimes or basking activity time did not influence the secondary sex ratio in these species (Foucart, Heulin, & Lourdais, 2018; Lorioux et al., 2013). Besides and to limit temperature differential between treatment, control group had permanent access to water at the opposite side of heat source (light bulb) and water was always sprayed out of thermoregulation time. Although our results clearly support the occurrence of WSD in early pregnant females in these two species, functional studies are now critically required to clarify the physiological pathways mediating WSD and notably interactions with maternal effects and hormonal regulation. Dehydration can cause physiological stress and endocrine dysregulations (Bradshaw, 1997; Dupoué et al., 2016), and we suspect WSD to be mediated by the impact of water restriction on stress‐mediated epigenetic regulation and maternal hormone mediation (Bowden, Ewert, & Nelson, 2000; Pieau & Dorizzi, 2004). In other animals, sex reversal is relatively frequent and potentially mediated with exogenous hormones (Stelkens & Wedekind, 2010). In reptiles species studied here, water could have reversed the sex of heterogametic females into males through an inhibition of aromatase activity early in development (Ramsey & Crews, 2009). Besides, in both species, dehydration induced an increase in mother corticosterone secretion (Dupoué et al., 2016, 2018), this hormone being known to interact with sex determination in other lizard species (Warner, Radder, & Shine, 2009). These proximate mechanisms should be clarified in future experiments and by more detailed investigations of the lizard karyotypes or genetic sex markers. Our results also showed that WSD is stage‐dependent since we found greater distortions of the secondary sex ratio when water restriction occurred earlier in gestation. As for the temperature sensitive period (Ramsey & Crews, 2009), this suggests that WSD is more likely to occur in early developmental stages before and/or during gonadal differentiation than in later development stages.

One evolutionary mechanism by which WSD is likely to be maintained involves sex‐specific effects of maternal water restriction on offspring fitness and therefore different fitness optima between sons and daughters (Charnov & Bull, 1977; Warner & Shine, 2008). Yet, the relationship between WSD and early fitness indicators showed either no differential optimum between juvenile females and males in vipers or complex year‐dependent sex‐specific growth trajectories in lizards. Early body growth was enhanced in neonate vipers from water‐deprived mothers (Dupoué et al., 2016) and this response was equivalent between juvenile females and males. In turn for lizards, juvenile female and male fitness differed between water restriction treatments and years. On one hand, juvenile males that faced water restriction during embryonic life had a lower early growth rate when exposed to a normal or an abnormally warm and dry summer conditions. On the other hand, maternal water restriction impacted the early growth trajectories of juvenile females only during a warm and wet summer. Eventually, these variation in growth rates were paralleled by annual variation of the survival rate although the negative impact of year and maternal dehydration on survival was similar between males and females. Together, these annual and sex‐specific responses are consistent with previous observations that male fitness curves are more sensitive to maternal dehydration and availability of free water in natural (Le Galliard et al., 2006) and seminatural or laboratory experiments (Dupoué et al., 2018; Romero‐Diaz, Breedveld, & Fitze, 2017). These findings however stand against conclusions of theoretical evolutionary models of ESD and sex reversal, where growth and survival of sons from water restricted females should be higher than those of daughters (Charnov & Bull, 1977; Pezaro et al., 2017; Warner & Shine, 2008). Since our results provide either no support for such sex‐specific fitness curves (i.e., vipers) or even the opposite tendency (i.e., lizards), the fitness benefits of WSD remain unclear so far. This suggests that WSD may not have any adaptive significance. Alternatively, one hypothesis, which remains to be tested, could be that WSD was selected in lizards to compensate for an average deleterious effects of dehydration stress on sons’ fitness (Conover & Van Voorhees, 1990). In support of this alternative hypothesis in lizards, the fitness of the minority sex from each treatment group was differently impacted by postnatal environments across the three study years. Specifically, sex differences in early growth rates were exacerbated when postnatal environment matched developmental conditions (daughters: control treatment followed by wet summers vs. sons: water restriction followed by summer drought). These results imply that consistent differences in habitat water availability may lead to a female‐biased adult sex ratio, which may select for individual strategies to produce the rarest sex according to Fisher's assumptions of frequency‐dependent selection models (Fisher, 1930).

Temperature usually covaries with water availability and both are critical determinants of phenotypic plasticity in ectotherms (Kearney & Porter, 2009). In the two species studied here, it was previously suggested that sex was determined solely by GSD, with females being the heterogametic sex. Here for the first time, we documented that one snake and one lizard species, GSD can be override by another environmental cue than temperature. Our study is still preliminary and both the proximate mechanisms and the adaptive significance of WSD remain to be elucidated. Taken the vital function of water, the considerable water embryonic demand during gestation and the convergent results for WSD in two distantly related species with distinct tolerance to water restriction, we predict that WSD in early gestation might represent a general pattern in reptiles (Freeman & Vitale, 1985; Sifuentes‐Romero et al., 2018). Water availability is expected to strongly change in the near future thereby questioning the interaction between WSD and population trends as for TSD (Pezaro et al., 2017). Our study thereby calls for general investigations of WSD and offers new insights and perspectives for our understanding on complex sex determination processes.

## CONFLICTS OF INTEREST

None declared.

## AUTHORS’ CONTRIBUTIONS

All authors conceived the ideas, methodology and contributed in acquiring the data. AD led the experiment conception, the analyses and the manuscript redaction together with OL and JFLG. All authors contributed critically to the manuscript draft and gave final accordance for publication.

## ETHICAL STATEMENT

The study on the Aspic viper was performed in accordance with laws relating to the capture, the transport and the welfare of the animals (DREAL#09/346/DEROG).

Studies on the Common lizard were performed in accordance with laws relating to the capture, the transport and the welfare of the animals (APAFIS#5108‐2016040811272391). Animal care and breeding was performed by authorized personnel under permit DTTP‐2008–449 issued to J‐F Le Galliard.

## Data Availability

Data available from the Dryad Digital Repository: https://doi.org/10.5061/dryad.mv06pv1.

## References

[ece35229-bib-0001] Aprea, G. , Gentilli, A. , Zuffi, M. A. L. , & Odierna, G. (2006). The karyology of Vipera aspis, V. atra, V. hugyi, and Cerastes vipera. Amphibia‐Reptilia, 27, 113–119.

[ece35229-bib-0002] Bachtrog, D. , Mank, J. E. , Peichel, C. L. , Kirkpatrick, M. , Otto, S. P. , Ashman, T.‐L. . … Vamosi J. C. . (2014). Sex determination: Why so many ways of doing it? PLoS Biology, 12, e1001899. 24983465 10.1371/journal.pbio.1001899 PMC4077654

[ece35229-bib-0003] Bolker, B. M. , Brooks, M. E. , Clark, C. J. , Geange, S. W. , Poulsen, J. R. , Stevens, M. H. H. , White J. S. . (2009). Generalized linear mixed models: A practical guide for ecology and evolution. Trends in Ecology & Evolution, 24, 127–135. 19185386 10.1016/j.tree.2008.10.008

[ece35229-bib-0004] Bonnet, X. , Lourdais, O. , Shine, R. , & Naulleau, G. (2002). Reproduction in a typical capital breeder: Costs, currencies, and complications in the aspic viper. Ecology, 83, 2124–2135.

[ece35229-bib-0005] Bonnet, X. , Naulleau, G. , & Shine, R. (2017). The evolutionary economics of embryonic‐sac fluids in squamate reptiles. American Naturalist, 189, 333–344. 10.1086/690119 28221829

[ece35229-bib-0006] Bowden, R. M. , Ewert, M. A. , & Nelson, C. E. (2000). Environmental sex determination in a reptile varies seasonally and with yolk hormones. Proceedings of the Royal Society B‐Biological Sciences, 267, 1745–1749. 10.1098/rspb.2000.1205 PMC1690737 12233772

[ece35229-bib-0007] Bradshaw, S. (1997). Homeostasis in desert reptiles. Berlin: Springer Verlag.

[ece35229-bib-0008] Capel, B. (2017). Vertebrate sex determination: Evolutionary plasticity of a fundamental switch. Nature Reviews. Genetics. 18: 675–689. Nature Publishing Group. 10.1038/nrg.2017.60 28804140

[ece35229-bib-0009] Charnov, E. L. , & Bull, J. J. (1977). When is sex environmentally determined? Nature, 267, 192–193. 10.1038/266828a0 865602

[ece35229-bib-0010] Chevalier, M. , Dufaure, J. P. , & Lecher, P. (1979). Cytogenetic study of several species of Lacerta (Lacertidae, Reptilia) with particular reference to sex chromosome. Genetica, 50, 11–18.

[ece35229-bib-0011] Conover, D. O. , & Van Voorhees, D. A. (1990). Evolution of a balanced sex ratio by frequency‐dependent selection in a fish. Science (80‐. ). 250: 1556–1558. 10.1126/science.250.4987.1556 17818284

[ece35229-bib-0012] Dupoué, A. , Angelier, F. , Brischoux, F. , DeNardo, D. F. , Trouve, C. , Parenteau, C. , …. Lourdais O. . (2016). Water deprivation increases maternal corticosterone levels and enhances offspring growth in the snake Vipera aspis. Journal of Experimental Biology, 219, 658–667. 26747902 10.1242/jeb.132639

[ece35229-bib-0013] Dupoué, A. , Brischoux, F. , Angelier, F. , DeNardo, D. F. , Wright, C. D. , & Lourdais, O. (2015). Intergenerational trade‐off for water may induce a mother‐offspring conflict in favour of embryos in a viviparous snake. Functional Ecology, 29, 414–422.

[ece35229-bib-0014] Dupoué, A. , Le Galliard, J. F. , Josserand, R. , DeNardo, D. F. , Decencière, B. , Agostini, S. , … Meylan S. . (2018). Water restriction causes an intergenerational trade‐off and delayed mother‐offspring conflict in a viviparous lizard. Functional Ecology, 32, 676–686.

[ece35229-bib-0015] Fisher, R. A. (1930). The genetical theory of natural selection. Oxford, UK: Oxford University Press.

[ece35229-bib-0016] Fitze, P. S. , & Le Galliard, J.‐F. (2008). Operational sex ratio, sexual conflict and the intensity of sexual selection. Ecology Letters, 11, 432–439. 18279355 10.1111/j.1461-0248.2008.01158.x

[ece35229-bib-0017] Foucart, T. , Heulin, B. , & Lourdais, O. (2018). Small changes, big benefits: Testing the significance of maternal thermoregulation in a lizard with extended egg retention (Zootoca vivipara). Biological Journal of the Linnean Society, 125, 280–291.

[ece35229-bib-0018] Freeman, D. C. , & Vitale, J. J. (1985). The influence of environment on the sex ratio and fitness of spinach. Botanical Gazette, 146, 137–142.

[ece35229-bib-0019] Holleley, C. E. , O'Meally, D. , Sarre, S. D. , Marshall Graves, J. A. , Ezaz, T. , Matsubara, K. , … Georges, A. . (2015). Sex reversal triggers the rapid transition from genetic to temperature‐dependent sex. Nature, 523, 79–82. 26135451 10.1038/nature14574

[ece35229-bib-0020] Kearney, M. , & Porter, W. (2009). Mechanistic niche modelling: Combining physiological and spatial data to predict species’ ranges. Ecology Letters, 12, 334–350. 19292794 10.1111/j.1461-0248.2008.01277.x

[ece35229-bib-0021] Kokko, H. , Klug, H. , & Jennions, M. D. (2012). Unifying cornerstones of sexual selection: Operational sex ratio, Bateman gradient and the scope for competitive investment. Ecology Letters, 15, 1340–1351. 22925080 10.1111/j.1461-0248.2012.01859.x

[ece35229-bib-0022] Kraak, S. B. M. , & Pen, I. (2002). Ses‐determining mechanisms in vertebrates. In I. C. W. Hardy (Ed.), Sex Ratios, Concepts and Research Methods (pp. 158–177). Cambridge, UK: Cambridge University Press.

[ece35229-bib-0023] Ladyman, M. , & Bradshaw, D. (2003). The influence of dehydration on the thermal preferences of the Western tiger snake Notechis Scutatus. Journal of Comparative Physiology, 173, 239–246. 12743727 10.1007/s00360-003-0328-x

[ece35229-bib-0024] Le Galliard, J. F. , Clobert, J. , & Ferrière, R. (2004). Physical performance and darwinian fitness in lizards. Nature, 432, 502–505. 15565154 10.1038/nature03057

[ece35229-bib-0025] Le Galliard, J. F. , Fitze, P. S. , Ferrière, R. , & Clobert, J. (2005). Sex ratio bias, male aggression, and population collapse in lizards. Proceedings of the National Academy of Sciences of the United States of America, 102, 18231–18236. 16322105 10.1073/pnas.0505172102 PMC1312374

[ece35229-bib-0026] Le Galliard, J.‐F. , Le Bris, M. , & Clobert, J. (2003). Timing of locomotor impairment and shift in thermal preferences during gravidity in a viviparous lizard. Functional Ecology, 17, 877–885.

[ece35229-bib-0027] Le Galliard, J.‐F. , Massot, M. , Landys, M. M. , Meylan, S. , & Clobert, J. (2006). Ontogenic sources of variation in sexual size dimorphism in a viviparous lizard. Journal of Evolutionary Biology, 19, 690–704. 16674566 10.1111/j.1420-9101.2006.01094.x

[ece35229-bib-0028] Lecomte, J. , Clobert, J. , & Massot, M. (1992). Sex identification in juveniles of Lacerta vivipara. Amphibia‐Reptilia, 13, 21–25.

[ece35229-bib-0029] Lorenzon, P. , Clobert, J. , Oppliger, A. , & John‐Alder, H. (1999). Effect of water constraint on growth rate, activity and body temperature of yearling common lizard (Lacerta vivipara). Oecologia, 118, 423–430. 28307409 10.1007/s004420050744

[ece35229-bib-0030] Lorioux, S. , Vaugoyeau, M. , DeNardo, D. F. , Clobert, J. , Guillon, M. , & Lourdais, O. (2013). Stage dependence of phenotypical and phenological maternal effects: Insight into squamate reptile reproductive strategies. American Naturalist, 182, 223–233. 10.1086/670809 23852356

[ece35229-bib-0031] Lourdais, O. , Lorioux, S. , Dupoué, A. , Wright, C. , & DeNardo, D. F. (2015). Embryonic water uptake during pregnancy is stage‐ and fecundity‐dependent in the snake Vipera aspis. Comparative Biochemistry and Physiology. Part A, Molecular & Integrative Physiology. 189: 102–106. Elsevier B.V. 10.1016/j.cbpa.2015.07.019 26255703

[ece35229-bib-0032] Mank, J. E. , & Uller, T. (2014). The Evolution of Sex Determination in Animals. In J. T. Streelman (Ed.), Advances in Evolutionary Developmental Biology, 1st ed. (pp. 15–36). Hoboken, NJ: John Wiley and Sons.

[ece35229-bib-0033] Matsumoto, Y. , Buemio, A. , Chu, R. , Vafaee, M. , & Crews, D. (2013). Epigenetic control of gonadal aromatase (cyp19a1) in temperature‐dependent sex determination of red‐eared slider turtles. PLoS ONE, 8, e63599. 23762231 10.1371/journal.pone.0063599 PMC3676416

[ece35229-bib-0034] Packard, G. C. (1991). The physiological and ecological importance of water to embryos of oviparous reptiles. In D. C. Deeming , & M. W. J. Ferguson (Eds.), Egg incubation: Its effect on embryonic development in birds and reptiles (pp. 213–228). Cambridge, UK: Cambridge University Press.

[ece35229-bib-0035] Pen, I. , Uller, T. , Feldmeyer, B. , Harts, A. , While, G. M. , & Wapstra, E. (2010). Climate‐driven population divergence in sex‐determining systems. Nature. 468: 436–438. Nature Publishing Group. 20981009 10.1038/nature09512

[ece35229-bib-0036] Pezaro, N. , Doody, J. S. , & Thompson, M. B. (2017). The ecology and evolution of temperature‐dependent reaction norms for sex determination in reptiles: A mechanistic conceptual model. Biological Reviews, 92, 1348–1364. 27296304 10.1111/brv.12285

[ece35229-bib-0037] Pieau, C. , & Dorizzi, M. (2004). Oestrogens and temperature‐dependent sex determination in reptiles: All is in the gonads. Journal of Endocrinology, 181, 367–377. 15171684 10.1677/joe.0.1810367

[ece35229-bib-0038] Quinn, A. E. , Georges, A. , Sarre, S. D. , Guarino, F. , Ezaz, T. , & Marshall Graves, J. A. (2007). Temperature sex reversal implies sex gene dosage in a reptile. Science (80‐. ). 316: 411. 10.1126/science.1135925 17446395

[ece35229-bib-0039] Ramsey, M. , & Crews, D. (2009). Steroid signaling and temperature‐dependent sex determination – Reviewing the evidence for early action of estrogen during ovarian determination in the red‐eared slider turtle. Seminars in Cell & Developmental Biology, 20, 283–292. 18992835 10.1016/j.semcdb.2008.10.004 PMC2695493

[ece35229-bib-0040] Robert, K. A. , & Thompson, M. B. (2001). Sex determination: Viviparous lizard selects sex of embryos. Nature, 412, 698–699. 11507628 10.1038/35089135

[ece35229-bib-0041] Romero‐Diaz, C. , Breedveld, M. C. , & Fitze, P. S. (2017). Climate effects on growth, body condition, and survival depend on the genetic characteristics of the population. American Naturalist, 190, 649–662. 10.1086/693780 29053364

[ece35229-bib-0042] Sifuentes‐Romero, I. , Tezak, B. M. , Milton, S. L. , & Wyneken, J. (2018). Hydric environmental effects on turtle development and sex ratio. Zoology. 126: 89–97. Elsevier. 29217120 10.1016/j.zool.2017.11.009

[ece35229-bib-0043] Stelkens, R. B. , & Wedekind, C. (2010). Environmental sex reversal, Trojan sex genes, and sex ratio adjustment: Conditions and population consequences. Molecular Ecology, 19, 627–646. 20088884 10.1111/j.1365-294X.2010.04526.x

[ece35229-bib-0044] Uller, T. , Pen, I. , Wapstra, E. , Beukeboom, L. W. , & Komdeur, J. (2007). The evolution of sex ratios and sex‐determining systems. Trends in Ecology & Evolution, 22, 292–297. 17418448 10.1016/j.tree.2007.03.008

[ece35229-bib-0045] Warner, D. A. , Radder, R. S. , & Shine, R. (2009). Corticosterone exposure during embryonic development affects offspring growth and sex ratios in opposing directions in two lizard species with environmental sex determination. Physiological and Biochemical Zoology, 82, 363–371. 19143534 10.1086/588491

[ece35229-bib-0046] Warner, D. A. , & Shine, R. (2008). The adaptive significance of temperature‐dependent sex determination in a reptile. Nature, 451, 566–568. 18204437 10.1038/nature06519

